# Supplementary material for: Identification of novel regulators of dendrite arborization using cell type-specific RNA metabolic labeling
Source: PLoS One. 2020 Dec 2;15(12):e0240386. doi: 10.1371/journal.pone.0240386 (PMC7710095; doi:10.1371/journal.pone.0240386)
Supplement: S1 Fig — (PDF) [file pone.0240386.s001.pdf]

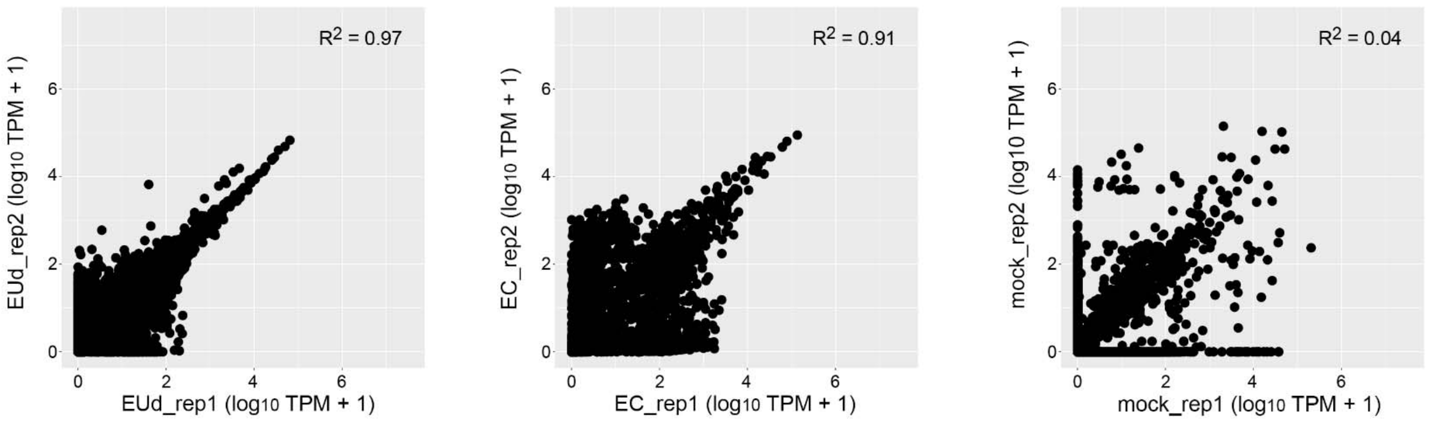

Supplemental Figure 1. Transcript level correlations for biological replicates. TPM = transcripts per million.
