# Supplementary material for: Identification of novel regulators of dendrite arborization using cell type-specific RNA metabolic labeling
Source: PLoS One. 2020 Dec 2;15(12):e0240386. doi: 10.1371/journal.pone.0240386 (PMC7710095; doi:10.1371/journal.pone.0240386)
Supplement: S2 Fig — (PDF) [file pone.0240386.s002.pdf]

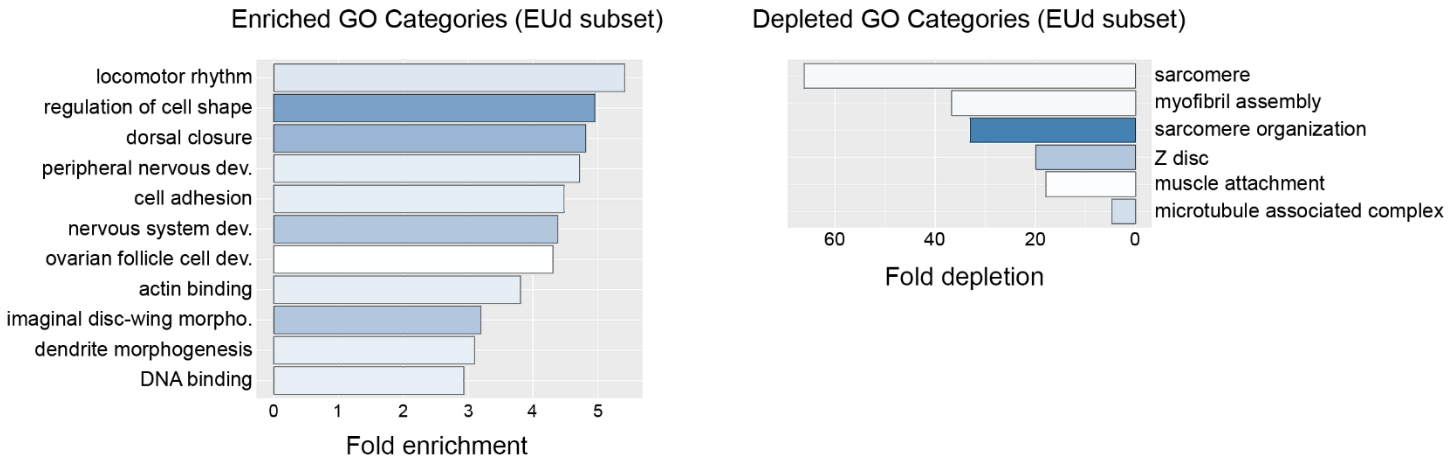

Supplemental Figure 2. Gene ontology of enriched and depleted transcripts based on EC-RNA compared to the subset ref-RNA. GO categories over-represented among EC-RNA enriched and depleted genes. Observed / expected value = frequency of category genes in EC-RNA / frequency in the *Drosophila* genome. Heatmap = Bonferroni-corrected P-values.
